# Digital papillary adenocarcinoma: A case report of a rare malignant tumour with recommendations on management and follow-up

**DOI:** 10.1016/j.ijscr.2025.110922

**Published:** 2025-01-21

**Authors:** Varanindu Mudduwa, Mohammad Goodarzi, Richard Chalmers, Haitham Khashaba

**Affiliations:** Department of Plastic and Reconstructive Surgery, University Hospital North Durham, Durham DH1 5TW, United Kingdom

**Keywords:** Digital papillary adenocarcinoma, Rare malignancy, Misdiagnosis, HPV42, Radical excision, Case report

## Abstract

**Introduction:**

Digital papillary adenocarcinoma (DPAC) is a rare malignant tumour of the sweat glands, usually in the digits. It has a high rate of recurrence and metastasis, yet there's a lack of guidelines for its diagnosis and management. Therefore, this report aims to evaluate procedures that provide the best outcomes, which will help create a consensus for its management.

**Case presentation:**

This case report presents a 47-year old male who had a painless hyperkeratotic patch on his left index finger, with an additional cystic lesion underlying it. This was diagnosed as a hidradenoma, which later changed to DPAC. His finger was amputated through the head of the middle phalanx. A positive sentinel lymph node biopsy led to a left axillary lymph node dissection, which revealed micrometastasis. The patient declined radiotherapy and was on a melanoma follow-up plan. To date there is no evidence of recurrence.

**Clinical discussion:**

Reviewing studies supported the use of immunohistochemical analysis to identify specific markers, especially HPV42. Sentinel lymph node biopsy and radical excision or amputation had the lowest rate of recurrence and thus should be common practice alongside long-term follow-up. Specific follow-up criteria are debated, yet this case may offer a solution by following the melanoma criteria.

**Conclusion:**

Histological and immunohistochemical analysis (including HPV42 detection), SLNB, and radical excision or amputation are optimal for DPAC management. Long-term follow-up, possibly using melanoma criteria, is crucial. Further research is needed to establish definitive guidelines.

## Highlights

• Digital papillary adenocarcinoma (DPAC) is a rare malignancy.
• DPAC often mimics benign cystic lesions leading to frequent misdiagnosis.
• There is a strong association between DPAC and HPV42 infection.
• Wide local excision or amputation is recommended over conservative surgery.
• This case following the melanoma follow-up protocol is unique.

## 1 Introduction

Digital papillary adenocarcinoma (DPAC) is a rare and aggressive malignant tumour originating from the eccrine sweat glands, predominantly in acral sites, particularly those of the digits [1]. With an incidence rate of 0.8 per 1,000,000 annually, DPAC is extremely rare and prone to misdiagnosis [1]. Despite its aggressive nature, DPAC presents as a firm, painless nodule, often mimicking a cystic lesion, contributing to difficulties in diagnosis [1]. Misdiagnosis can prove disastrous, given its high metastatic potential, with metastatic rates reported to be as high as 41 % [2]. Therefore, accurate diagnosis followed by timely and appropriate treatment is vital in optimising patient outcomes [3].

There are currently no guidelines for the diagnosis, treatment, or follow-up of DPAC. Given its rarity, every documented case of DPAC adds valuable insight that can aid in developing more effective diagnostic and therapeutic protocols. This case report presents a 47-year-old male with DPAC with the aim of evaluating an informed approach to its management and recommending management and follow-up as per the UK NICE (2022) melanoma guidelines [4]. The work has been reported in line with the SCARE and PROCESS criteria [5,6].

## 2 Case presentation

A 47-year-old male patient was referred by his general practitioner to the urgent Plastic Surgery clinic with a 3-month history of developing a small painless lump on his left index finger over the site of a previous burn scar from his adolescence. The patient reported no pain, tenderness, discharge, or bleeding from this lesion. His past medical history was cardiac ablation for Wolf Parkinson White syndrome around 20 years ago and obesity (with a BMI of 36). The patient was an ex-smoker.

**Fig. 1:**
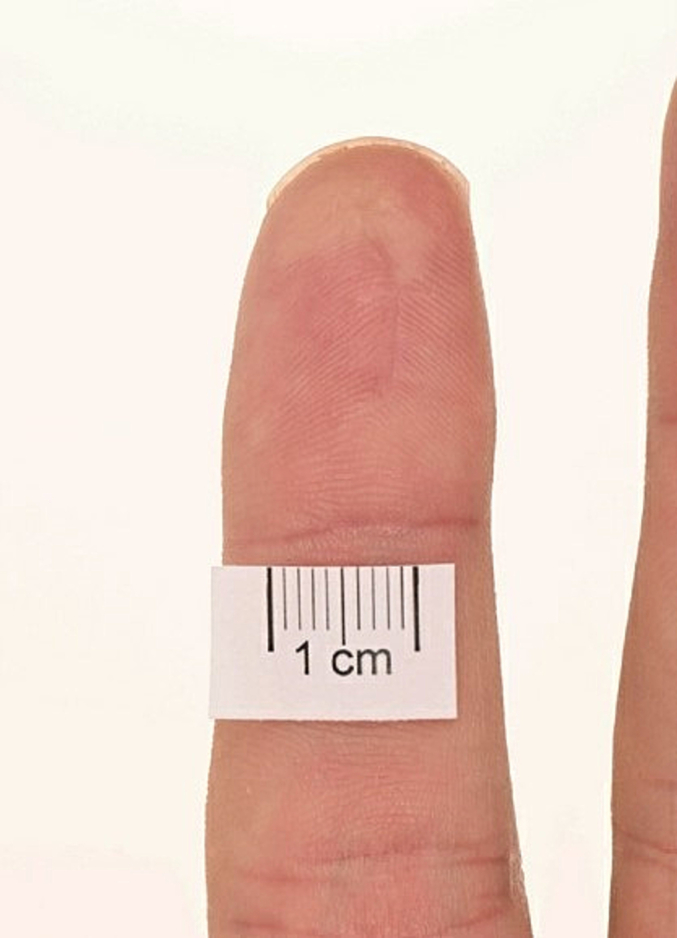
Pre-operative appearance of finger pulp.

**Fig. 2:**
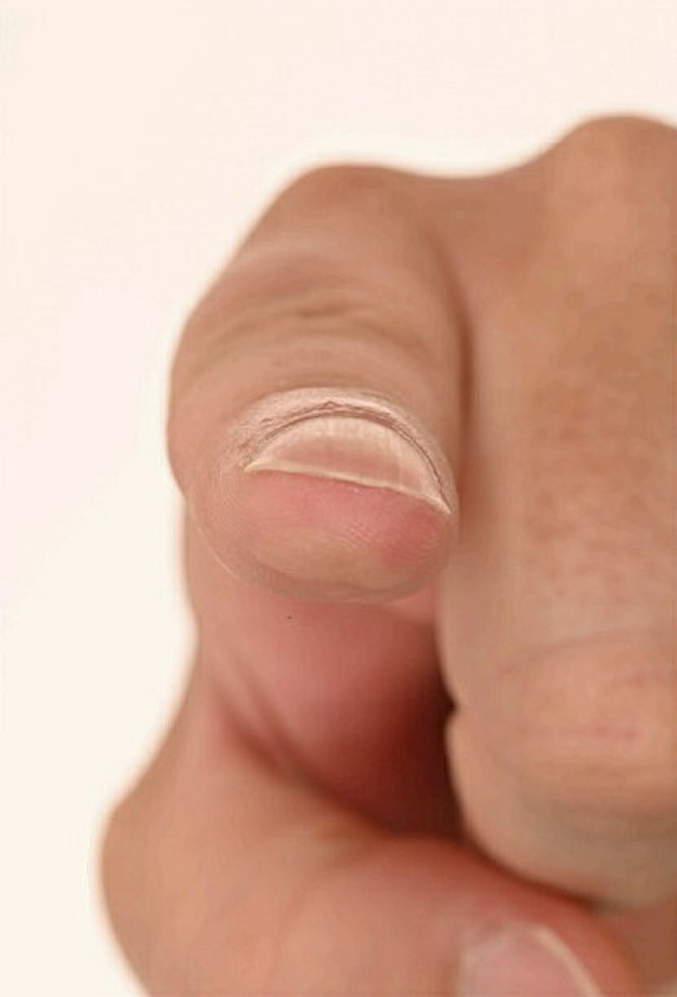
Pre-operative appearance of finger pulp.

Examination revealed a roughly 2 × 1 cm mobile, hyperkeratotic papule with exaggerated skin markings on the pulp space (shown in Fig. 1, Fig. 2). Additionally, an underlying, slightly round cystic lesion could be palpated. There was no lymphadenopathy. An excisional biopsy was carried out under local anaesthetic on the index finger lesion and initial histological analysis reported a benign cystic hidradenoma. This case was discussed at the skin MDT and further histological analysis revealed cellular areas of glandular and papillary proliferation protruding into the cystic spaces and multinucleated giant cells, which are characteristic histologic features of DPAC [2]. The degree of cystic changes observed was unusual for DPAC; however, further immunohistochemical analysis revealed EMA, CK, SMA, P63 and S-100 positivity. These findings were in keeping with DPAC and the diagnosis was appropriately changed by the pathology team [7,8].

**Fig. 3:**
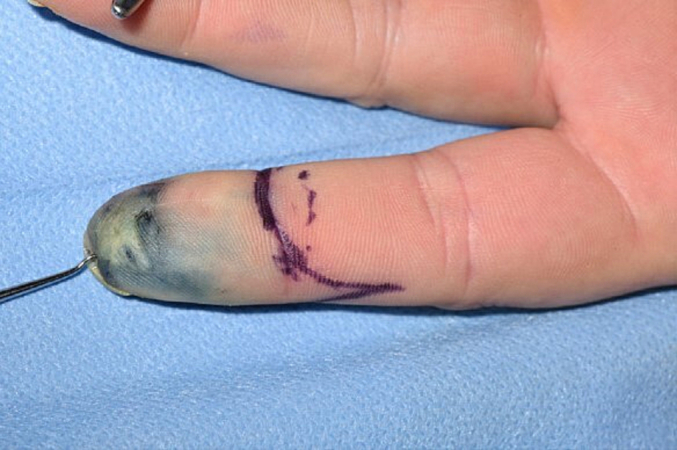
Intra-operative excision markings (note blue staining from Patent V dye injection to localise sentinel node).

**Fig. 4:**
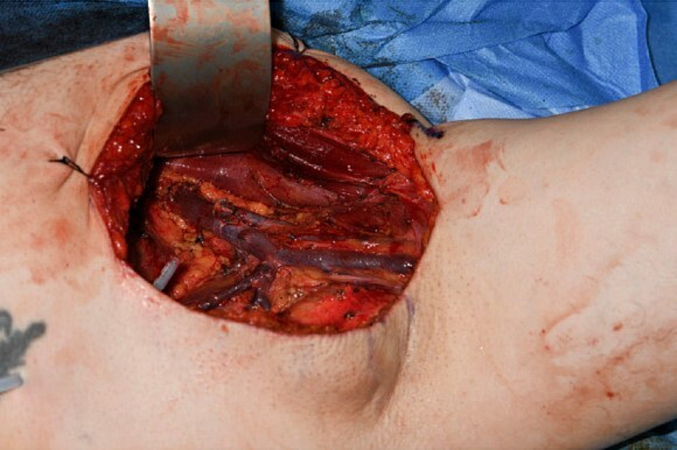
Intra-operative appearance of axillary lymphadenectomy.

Given the significant metastatic potential of DPAC, particularly to the lungs and regional lymph nodes, a staging computed tomography (CT) scan of the head, neck, chest, abdomen, and pelvis with contrast was performed [3] and demonstrated no evidence of metastatic disease. Following this, the patient underwent a 1 cm wide local excision, involving an amputation through the head of the middle phalanx and sentinel lymph node biopsy of the left axilla under general anaesthetic (shown in Fig. 3 – blue staining from Patent Blue V injection to allow localisation of sentinel lymph node). Initially, histological analysis of the sentinel lymph node and the left index finger did not demonstrate any evidence of malignancy. However, further immunohistochemistry assessment confirmed a positive sentinel lymph node biopsy. A block dissection of the left axillary lymph nodes was subsequently carried out under general anaesthetic (shown in Fig. 4). This demonstrated evidence of micrometastasis in one lymph node with clear margins. Postoperative adjuvant radiotherapy was offered but the patient declined due to concerns regarding adverse effects.

**Fig. 5:**
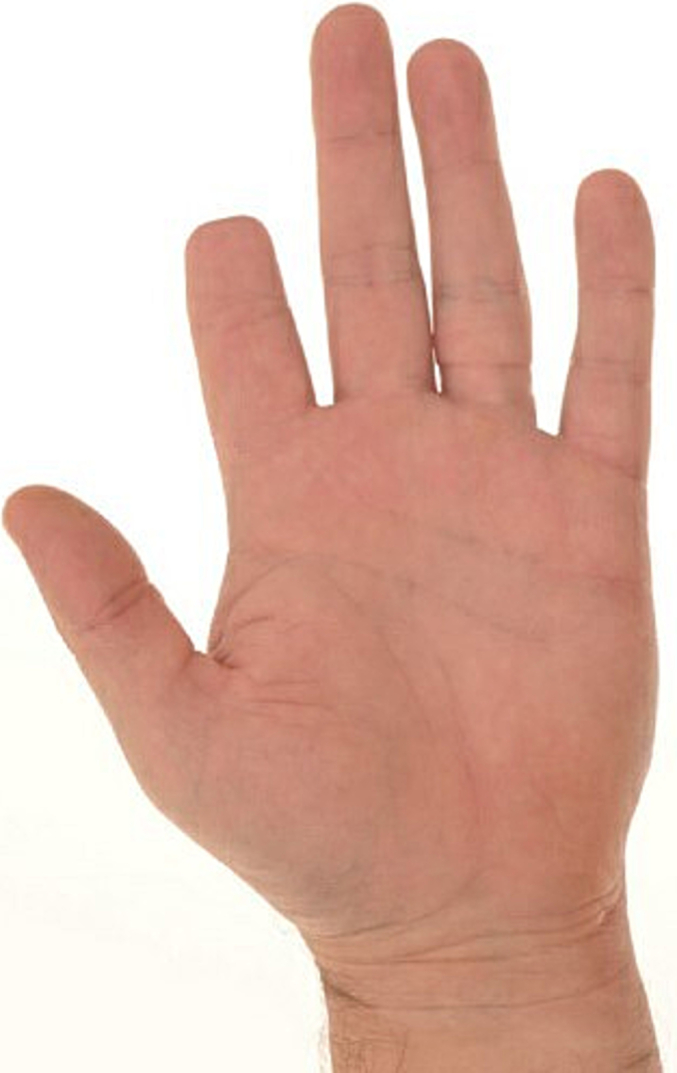
Post-operative healed appearance 6 months after excision.

With the lack of follow-up guidance of DPAC specifically, it was decided by the skin MDT to carry out follow-up as a Stage 3 melanoma using the NICE 2022 guideline [4]. This involves: 3 monthly clinic reviews with 6 monthly whole-body and brain contrast CT scans for 3 years followed by 6 monthly clinic reviews with yearly whole-body and brain contrast CT scans for 2 years. At his most recent follow-up, there were no new lesions or concerning features observed under dermoscopy, and no lymphadenopathy was noted indicating no recurrence or metastasis 18 months post-operatively (shown in Fig. 5).

## 3 Discussion

Digital papillary adenocarcinoma (DPAC) was first described by Kao and Helwig in 1987 as an “aggressive digital papillary tumour” due to its locally aggressive behaviour and tendency to occur on digits [2]. Unfortunately, despite more than four decades since its initial description, diagnosing DPAC remains challenging, and there is remains a lack of robust, evidence-based recommendations for excision margins, sentinel lymph node biopsy (SLNB), radiological staging investigations, and follow-up surveillance [9].

DPAC presents as a slow-growing, solitary, non-tender mass that arise from the fingers and toes, most frequently affecting the distal part of the finger or thumb [10]. They occur predominantly in men, with a 9:1 male-to-female ratio, and are most common in the 5th and 6th decades of life [11]. This slow growth pattern, coupled with the rarity of DPAC, often leads to clinical misdiagnosis as benign pathologies such as calluses, ganglion cysts, gout, or soft tissue infections [12].

The histological appearance of DPAC is distinctive consisting of solid cystic patterns of growth with regions of papillary extensions in cystic spaces in ductal and tubuloalveolar structures [2]. Despite these defining features, its rarity and similarity to other benign tumors have led to instances of misdiagnosis, with DPAC sometimes being incorrectly identified as acrospiroma [13] or spiradenoma [9].

In the presented case, initial an incorrect histological diagnosis of hidradenomas was made before immunohistochemistry analysis allowed correct the diagnosis to DPAC. Both DPAC and hidradenomas share well-defined dermal development patterns with tubular, cystic, and solid regions that have a high mitotic rate [7]. The histological diagnosis of DPAC can be refined using markers such as EMA, CK, P63, and SMA [8,14]. Wiedemeyer et al. emphasize that the presence of P63 and the absence of SMA and S100 in tumour cells are indicative of hidradenomas rather than DPAC [7]. Additionally, 96 % of DPAC cases contain viral DNA from HPV42, suggesting a causal link, whereas HPV42 is not detected in hidradenomas [15]. Therefore, combining histological and immunohistochemical analysis, including HPV42 detection, could reduce DPAC misdiagnosis and improve differentiation from other tumors.

The current consensus for treating DPAC is surgical intervention, with options including amputation or wide local excision [1]. Evidence indicates that radical excision is preferable to wide local excision, with recurrence rates of 57 % for wide local excision compared to 5 % for radical excision [2,3] These findings justify the need for urgent radical surgical intervention in order to maximise patient outcomes. However, a study of 19 cases, including seven treated with conservative surgical management (conservative excision or conservative surgical re-excision, without amputation) rather than amputation, found no higher recurrence rates with conservative surgical approaches [16]. Despite this, conservative treatment lacks sufficient evidence and is not recommended due to the aggressive nature of DPAC. There is currently no effective treatment for extensive metastasis, and both radiotherapy and chemotherapy lack supporting evidence [3].

Given the potential of DPAC to metastasise to the lymph nodes, SLNB is commonly performed during the staging [2]. In a single-centre study of 18 patients treated for DPAC with wide excision and SLNB identified positive nodal metastases in three of patients (none of whom had clinically palpable lymphadenopathy). These patients subsequently underwent completion lymph node dissection. At a median follow-up of 53 months, no individuals with negative SLNB had evidence of recurrence or metastases suggesting a significant correlation between a positive SLNB and recurrence/metastasis [17].

However, the therapeutic benefit of subsequent lymph node dissections remains unproven and there are no effective treatments currently for metastatic DPAC, questioning the purpose of SLNB [3]. Despite this, parallels with melanoma—where patients with positive SLNB who undergo complete lymphadenectomy show better outcomes—suggest that SLNB with lymph node dissection could offer prognostic and therapeutic benefits for DPAC patients [18,19]. More data is needed to confirm this.

Unlike other malignancies, histological and immunohistochemical features do not predict the risk of metastasis or recurrence in DPAC, necessitating long-term follow-up [1]. No standardized criteria exist, but many follow a variation of Kao et al.'s [2] protocol, involving annual clinical examinations and chest x-rays for ten years. This case following the melanoma follow-up protocol is unique and therefore, could be a point of reference for future cases.

## 4 Conclusion

The management of DPAC is an area of high speculation due to the small number of cases and studies available in the literature. Yet there are common findings throughout several cases which can help build a consensus. The combined use of histological and immunohistochemical analysis, with particular emphasis on detecting HPV42 may help prevent misdiagnosis. The use of SLNB and radical excision or amputation appears to be the most optimal management plan at present, alongside long-term follow-up. The specific follow-up criteria are still up for debate, but this case may offer a solution by following the melanoma criteria.

## Ethical approval

As this is a case report, it is exempt from ethical approval.

## Guarantor

HK is the author guarantor.

## Consent for publication

Written informed consent was obtained from the patient for publication of this case report and accompanying images. A copy of the written consent is available for review by the Editor-in-Chief of this journal on request.

## Funding

None.

## Author contribution

VM and MG should be considered as joint first-authors as both contributed equally to this paper. HK was responsible for the study's conception and design. Material preparation and data collection were performed by VM, MG, and RC. The first draft of the manuscript was written by VM and MG. and all authors commented on previous versions of the manuscript. All authors read and approved the final manuscript.

## Conflict of interest statement

No conflict of interest to declare from all authors.
